# CCR5 promoter activity correlates with HIV disease progression by regulating CCR5 cell surface expression and CD4 T cell apoptosis

**DOI:** 10.1038/s41598-017-00192-x

**Published:** 2017-03-22

**Authors:** Anjali Joshi, Erin B. Punke, Melina Sedano, Bethany Beauchamp, Rima Patel, Cassady Hossenlopp, Ogechika K. Alozie, Jayanta Gupta, Debabrata Mukherjee, Himanshu Garg

**Affiliations:** 1 Center of Emphasis in Infectious Diseases, Texas Tech University Health Sciences Center, El Paso, TX USA; 2 Paul L Foster School of Medicine, Texas Tech University Health Sciences Center, El Paso, TX USA; 3 Department of Internal Medicine, Texas Tech University Health Sciences Center, El Paso, TX USA; 4 Department of Biomedical Sciences, Texas Tech University Health Sciences Center, El Paso, TX USA; 5 Department of Health Sciences, College of Health Professions and Social Work, Florida Gulf Coast University, Fort Myers, FL USA

## Abstract

CCR5 is the major co-receptor for HIV and polymorphisms in the CCR5 gene as well as promoter region that alter cell surface expression have been associated with disease progression. We determined the relationship between CCR5 promoter polymorphisms and CD4 decline and other immunopathological features like immune activation and CD4+ T cell apoptosis in HIV patients. CCR5 promoter haplotype HHC was significantly associated with higher CD4 counts in patients. The relative promoter activity (RPA) of each haplotype was determined *in vitro* and combined promoter activity based on both alleles (CRPA) was assigned to each patients. Interestingly, CCR5 CRPA correlated inversely with CD4 counts and CD4:CD8 ratio specifically in viremic patients. In normal individuals, the CRPA correlated with the number of CCR5+ CD4+ T cells in the peripheral blood suggesting an effect on CCR5 expression. In a subset of high viremic patients harboring R5 tropic HIV, there was a strong correlation between CCR5 CRPA and both CD4 counts and CD4 T cell apoptosis. Our study demonstrates that, CCR5 promoter polymorphisms correlate with CD4 T cell loss possibly by regulating CD4 T cell apoptosis in HIV patients. Furthermore, assigning CRPAs to each patient is a new method of translating genotype to phenotype.

## Introduction

CCR5 is a major co-receptor for HIV and has been known to play a significant role in HIV infection and pathogenesis. Polymorphisms in CCR5 gene and promoter region have been extensively studied and a correlation between CCR5 polymorphisms and HIV disease has been demonstrated by several groups^1–7^. However, it remains unclear as to how polymorphisms in the CCR5 gene and promoter region affect HIV disease progression.

The role of CCR5 in HIV disease was first recognized by the resistance of CCR5Δ32 homozygous individuals to HIV infection due to a lack of cell surface CCR5 protein^8, 9^. A 32 base pair deletion resulting in a truncated CCR5 protein that remains cytosolic was identified as the mechanism behind this resistance^10^. However, subsequent studies found that individuals heterozygous for CCR5Δ32, were susceptible to HIV infection, but showed a delayed progression to AIDS^10, 11^. The CCR5Δ32 heterozygous individuals have lower levels of CCR5 on their cell surface^12, 13^, which has been suggested as the mechanism behind the slower disease progression^14^.

Additionally, multiple single nucleotide polymorphisms have also been identified in the CCR5 promoter region that have been shown to be associated with HIV disease progression^2, 4, 15^. Collectively, these SNPs make up 8 CCR5 promoter haplotypes (HHA-HHG)^6^. Recent studies have shown that certain haplotypes like HHC are associated with slower progression to AIDS in the Caucasian population^6^. Studies elucidating the molecular mechanism have shown that the haplotypes have different promoter activity that also correlates with cell surface CCR5 expression^5, 16^. Thus, the protective effect of CCR5 promoter haplotypes on AIDS progression may largely be associated with reduction in cell surface CCR5 levels. Extensive population based studies have suggested a protective effect of some CCR5 promoter polymorphisms in terms of HIV acquisition^7, 17^. Although, most studies suggest that the protective effect of CCR5 polymorphisms may largely be limited to progression to AIDS in HIV+ individuals, the precise mechanism underlying the role of CCR5 levels in HIV disease remains unclear.

In this study, we took a different approach to understanding the potential role of CCR5 polymorphisms in clinical manifestations of HIV infection. We genotyped the CCR5 promoter region of HIV+ patients and determined the haplotypes for each allele. We then assessed the activity of each CCR5 promoter haplotype using an *in vitro* gene reporter assay. After normalizing the promoter activities to the ancestral HHA haplotype^6^, we assigned each haplotype a numerical value termed Relative Promoter Activity (RPA). We then calculated the Combined Relative Promoter Activity (CRPA) for each patient by adding the RPAs of each allele. As a result, we could convert the genotypic information into a numerical value that could be used to correlate with various clinical manifestations in HIV patients. We find that CRPA correlates with CD4 counts, AIDS phenotype and CD4 T cell apoptosis in HIV patients. In normal individuals CRPA correlates with CCR5+ cells in peripheral blood confirming a direct relationship with CCR5 expression. This indicates that CCR5 promoter polymorphisms alter CCR5 expression levels that influence CD4 apoptosis and consequently CD4 decline in HIV patients. To the best of our knowledge, this is the first study demonstrating a new approach of assigning numerical scores (CRPA) based on CCR5 haplotype that also correlates with HIV disease progression/pathogenesis.

## Results

### CCR5 promoter polymorphisms in HIV infected population

CCR5 promoter polymorphisms have been associated with HIV disease progression^2, 4–6^. Seven SNPs in the promoter region have been identified in the human population that collectively make up the haplotypes HHA through HHG (Supplementary Table 1). A 32 bp deletion in the CCR5 gene is also prominent in the human population and differentiates the HHG1 and HHG2 haplotypes^18^. We determined the SNPs in the CCR5 promoter region as well as the CCR5Δ32 genotype and the allele frequency of each haplotype in our HIV infected and control population (Table 1). The most frequent haplotypes in HIV infected individuals were HHC (42%) and HHE (28%) which collectively made up 70% of the total haplotypes. Our data is much in line with other studies showing that in terms of HIV infection, no particular SNP or haplotype was associated with acquisition of HIV. In fact, we found 3/50 (6%) of the patients in our study were CCR5Δ32 heterozygous which corresponds with the frequency of the mutation in Hispanic population^19^. We did not find CCR5Δ32 homozygous individuals in our HIV+ group, which is consistent with resistance of this population to HIV infection^8, 9^. Overall, our data indicate that our study population shows similar distribution of genotypes as seen in other Hispanic populations and CCR5 promoter polymorphism in our study population does not protect against acquisition of HIV infection.

**Table 1:** Frequency of different CCR5 promoter haplotypes in the HIV+ population and normal controls.

	Population (frequency)	
Haplotype	HIV+ (N = 50)	Normal (N = 28)
HHA	4 (0.040)	3 (0.054)
HHB	0 (0.000)	0 (0.000)
HHC	42 (0.420)	19 (0.339)
HHD	2 (0.020)	0 (0.000)
HHE	28 (0.280)	14 (0.250)
HHF	11 (0.110)	14 (0.250)
HHG1	10 (0.10)	4 (0.07)
HHG2	3 (0.03)	2 (0.035)

The HHG2 haplotype includes CCR5Δ32 genotype.

### Individual CCR5 promoter SNPs are associated with CD4 levels in HIV infected patients

**Figure 1:**
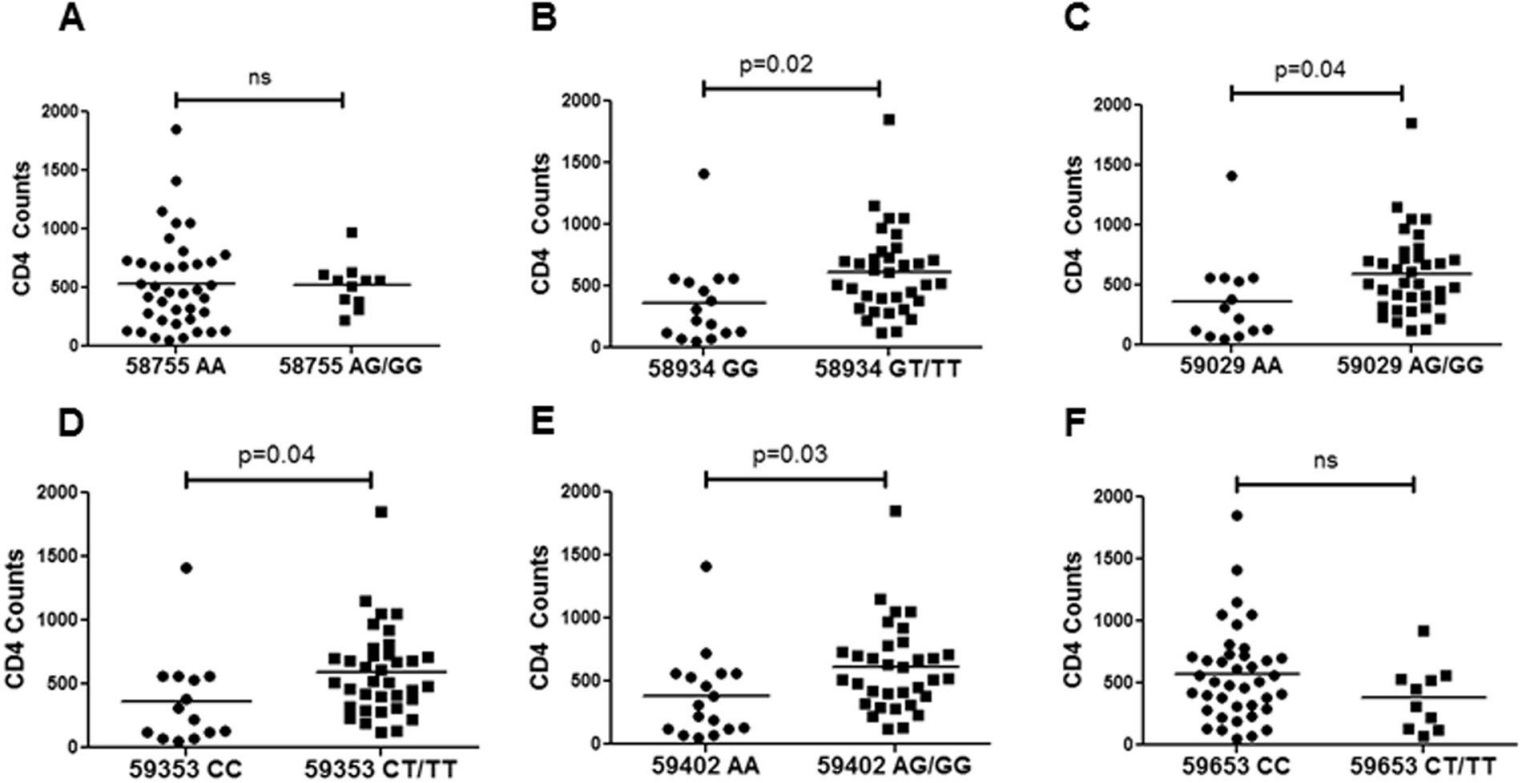
Association of SNPs in the CCR5 promoter with CD4 counts in HIV infected patients. Different SNPs in the CCR5 promoter region were determined by PCR amplification followed by sequencing using specific primers. Association of specific SNPs **(A)** 58755A (**B**) 58934G, (**C**) 59029A, (**D**) 59353C, (**E**) 59402A and (**F**) 59653C with CD4 counts in HIV infected individuals is shown. Two tailed student's t-test was used for statistical analysis.

Multiple studies have reported CCR5 promoter polymorphisms to be associated with disease progression and CD4 levels in patients^6, 20–22^. We determined whether specific SNPs correlated with CD4 levels in HIV infected individuals. We studied 6 SNPs: 58755A, 58934G, 59029A, 59353C, 59402 A and 59653C. Frequency of SNPs in the HIV+ and normal population is listed in Supplementary Table 2. Interestingly, we found that 58934GG (p = 0.02), 59029AA (p = 0.04), 59353CC (p = 0.04) and 59402AA (p = 0.03) were significantly associated with lower CD4 counts in our patient population (Fig. 1A–F). Closer analysis revealed that these SNPs collectively make up the non HHC group (Supplementary Table 1). Thus, our preliminary data analysis revealed that certain CCR5 promoter SNPs may play a role in regulating HIV disease progression as evident from the CD4 counts.

### HHC haplotype is associated with higher CD4 counts in HIV infected patients

**Figure 2:**
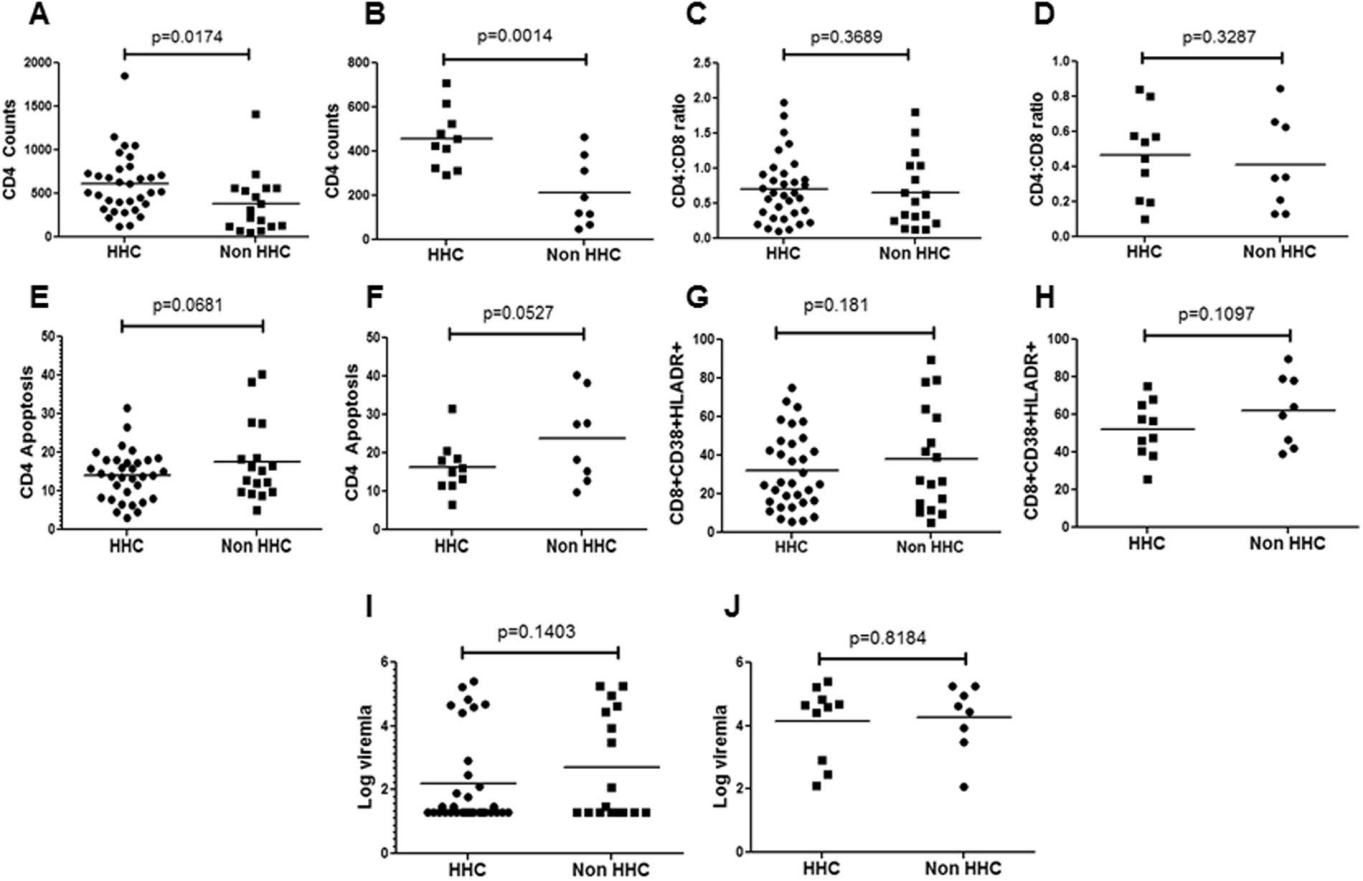
HHC haplotype is associated with higher CD4 counts in HIV infected patients. Based on CCR5 promoter haplotype determination, the HIV patient population was divided into HHC group, where at least one allele was HHC, or non HHC, where neither allele was HHC. CD4 counts in HHC versus non HHC groups in (**A**) all HIV+ and (**B**) viremic patients (>100 copies/ml viral RNA). CD4:CD8 ratio in HHC versus non HHC CCR5 groups in (**C**) all HIV+ and (**D**) viremic patients. CD4 apoptosis (active Caspase+) in HHC versus non HHC groups in (**E**) all HIV+ and (**F**) viremic patients. Immune activation defined by the presence of CD8+CD38+ HLADR+ cells in HHC versus non HHC CCR5 groups in (**G**) all HIV+ and (**H**) viremic patients. Differences in log viremia between HHC and non HHC groups in (**I**) all HIV+ and (**J**) viremic patients. One tailed student's t-test was used for statistical analysis.

Previous studies have reported a protective effect of HHC haplotype on the decline in CD4 counts in Caucasian population^6^. Our individual SNP analysis also suggested that HHC haplotype may be associated with higher CD4 levels (Fig. 1). We hence divided our population into HHC group (N = 33), where at least one allele was HHC, or non HHC group (N = 17), where neither allele was HHC (Supplementary Table 3). We then asked whether HHC versus non HHC haplotype affected the immunopathological features of HIV infection, including CD4 counts, immune activation, CD4 apoptosis and viremia. Interestingly, we found that CD4 counts in the non HHC group were lower than HHC group (p = 0.0174) (Fig. 2A), and this effect was more dramatic in viremic patients (viremic defined as ≥100 virus copies/ml) (p = 0.0014) (Fig. 2B) (Supplementary Table 4). Analysis of CD4:CD8 ratios, another predictor of HIV pathogenesis, did not show significant differences between HHC and non HHC groups in either all HIV+ (Fig. 2C) or viremic (Fig. 2D) HIV patients. Similarly, we also looked at CD4 apoptosis (defined as active caspase + cells) between the groups and found that non HHC group showed modestly higher CD4 apoptosis (Fig. 2E) that trended but was not statistically significant (p = 0.0681) in the HIV+ population. However, within the viremic group, there was a stronger trend towards higher apoptosis in non HHC group compared to HHC (p = 0.0527) (Fig. 2F). Immune activation, defined by the presence of CD8+CD38+HLADR+ cells is a classical immunopathological feature of HIV infection and correlates strongly with CD4 decline^23–25^. We hence looked at immune activation in these groups and found no significant difference in immune activation in CD8+ cells in the entire HIV+ group (p = 0.181) (Fig. 2G), or viremic patients (p = 0.1097) (Fig. 2H). With regards to log viremia, no significant differences were observed between the HHC and non HHC group in either the HIV+ (Fig. 2I) or viremic patients (Fig. 2J). Based on these data we can conclude that non HHC haplotype is associated with lower CD4 counts, which in part may be related to higher CD4 apoptosis in this group. The lack of difference in viremia in the two groups also indicates that in our study the effect of CCR5 polymorphism is likely to be independent of viremia.

### CCR5 promoter haplotypes have different transcriptional activity

**Figure 3:**
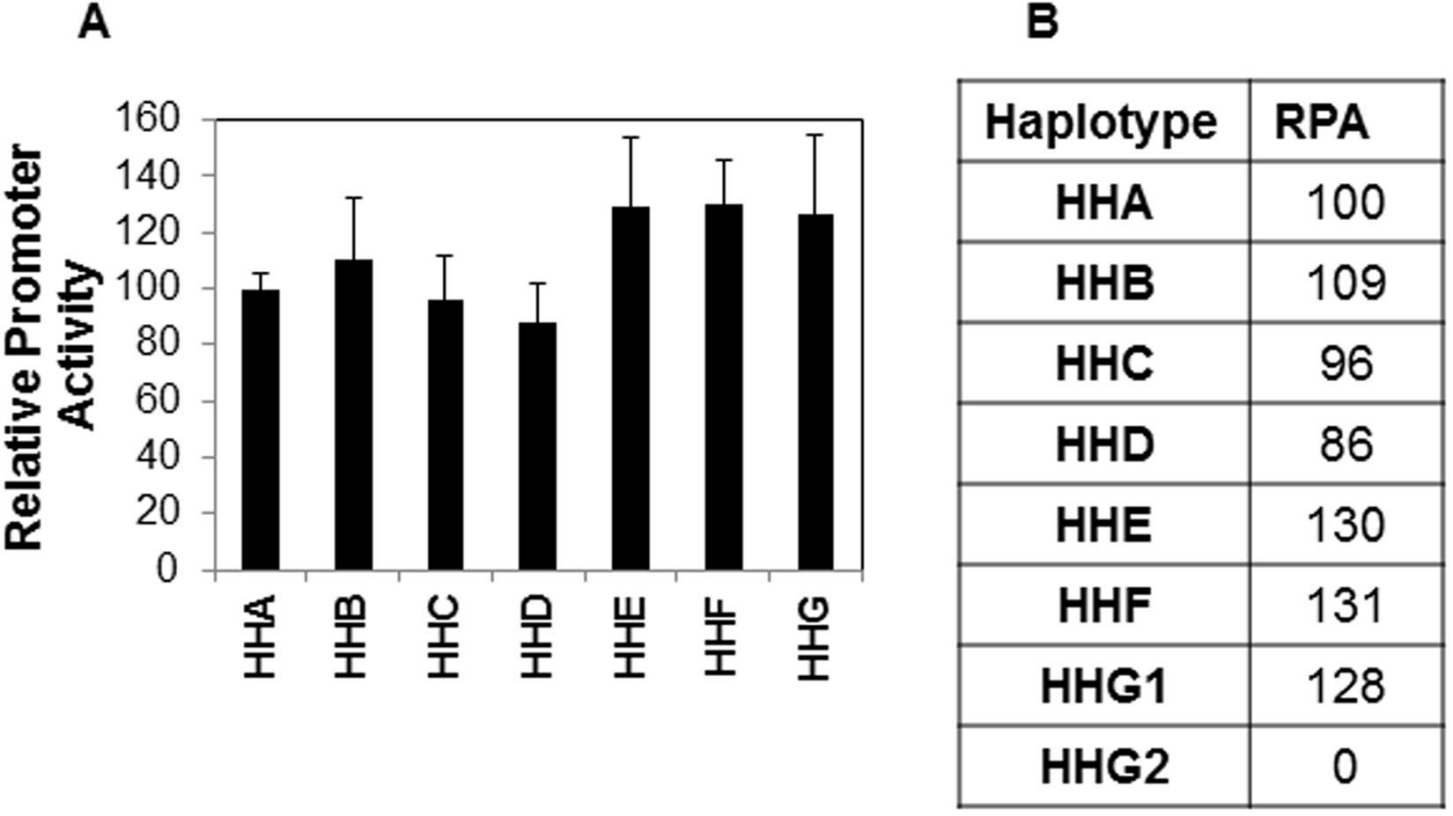
Transcriptional activity of different CCR5 promoter haplotypes. (**A**) 293 T cells were transfected with plasmid DNA containing CCR5 gene promoter haplotype (HHA, HHB, HHC, HHD, HHE, HHF and HHG) upstream of the luciferase gene. Promoter activity was determined 48 h later by measuring luciferase activity in cell lysates. Data was normalized to HHA, the ancestral haplotype. Bars represent mean ± SD of triplicate observations from 4 independent experiments. (**B**) Relative promoter activity (RPA) of each CCR5 promoter haplotype after normalizing to the ancestral allele HHA.

CCR5 promoter polymorphisms potentially modify gene expression and consequently affect cell surface expression of CCR5^3, 26^. As CCR5 is the major co-receptor for HIV and interactions between CCR5 and Env glycoprotein of HIV are believed to be fundamental to HIV infection, replication and HIV pathogenesis. Thus alterations in CCR5 levels are functionally significant to HIV disease. We have previously demonstrated that the levels of CCR5 on the cell surface can in fact alter bystander apoptosis mediated by HIV Env glycoprotein^27^. Hence, we next determined the promoter activity of each of the seven CCR5 promoter haplotypes (HHA-HHG) using a luciferase based gene reporter assay (Fig. 3A). We then normalized the activity of each promoter haplotype to the ancestral HHA haplotype. Based on these data, each haplotype was assigned a numerical value or the Relative Promoter Activity (RPA) (Fig. 3B). We found that the haplotypes could be divided into two groups. The low group included HHA, HHB, HHC and HHD while the high group comprised of HHE, HHF and HHG. Interestingly, the high group showed almost 30% higher RPA in our assay than HHA while HHC haplotype showed a low RPA of 96% (Fig. 3B). We assigned HHG2, which is in complete linkage disequilibrium with CCR5Δ32, a RPA value of 0 as the 32 bp deletion in CCR5 ORF results in a truncated protein that is not expressed on the cell surface^10^. Using this approach we could now assign each patient a Combined Relative Promoter Activity (CRPA) score based on the sum of the RPA of each allele. The CRPA of the common allele pairs in our study is shown in Supplementary Table 3. The CRPA score now provides us the opportunity of assigning a numerical value to the CCR5 genotype that can be correlated with different immunopathological parameters in HIV infection. Interestingly, we found that all the three CCR5Δ32 individuals in our HIV infected population had a high RPA allele (HHG1, HHE or HHF) as the second allele (Supplementary Table 3). This is interesting, as in the context of CCR5Δ32, the protective effect in heterozygous populations against HIV acquisition may be related to the RPA of the second allele, as has been reported by Hladik *et al*.^3^. Similar luciferase based gene reporter assays were also conducted after cloning a smaller version of the CCR5 promoter as described by Mummidi *et al*.^5^. Data with smaller CCR5 promoter yielded similar Relative Promoter Activity (Supplementary Figure 1).

### Combined Relative promoter activity of both alleles in HIV patients as a new parameter for disease progression

**Figure 4:**
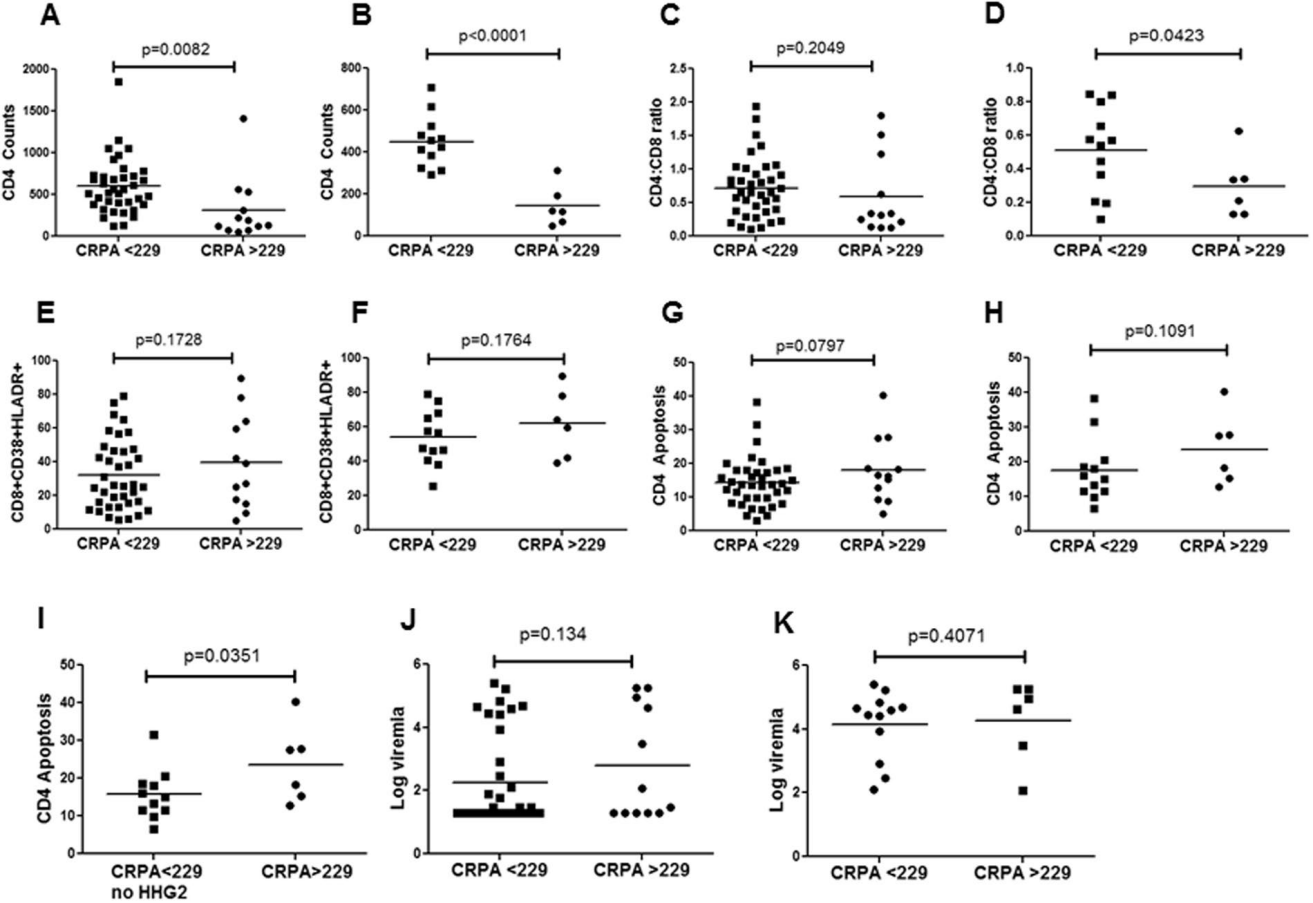
Combined CCR5 relative promoter activity (CRPA) of both alleles in HIV patients correlates with CD4 counts. Each patient was assigned a numerical CRPA score based on the RPA of both alleles. The patient population was then divided into two groups of CRPA > 229 or CRPA < 229. CD4 counts in CRPA > 229 vs CRPA < 229 in (**A**) all HIV+ patients (**B**) viremic patients. CD4:CD8 ratio in CRPA > 229 vs CRPA < 229 in (**C**) all HIV+ patients (**D**) viremic patients. CD8 immune activation in CRPA > 229 vs CRPA < 229 in (**E**) all HIV+ patients (**F**) viremic patients. CD4 apoptosis in CRPA > 229 vs CRPA < 229 in (**G**) all HIV+ patients (**H**) viremic patients. (**I**) CD4 apoptosis in CRPA > 229 vs CRPA < 229 no HHG2 (after removing one data point from an HHG2 patient). Log viremia in CRPA > 229 vs CRPA < 229 in (**J**) all HIV+ or (**K**) viremic patients. One tailed student's t-test was used for statistical analysis.

Using the above methods we assigned each patient a numerical value or CRPA score for CCR5 promoter activity and then asked whether CRPA of each patient correlated with CD4 counts and other immunopathological parameters. Based on CRPA score, our population could be divided into two groups of CRPA >229 or CRPA <229. This classification was based on the observation that >229 did not contain any HHC haplotype (Supplementary Table 3). Once again, we found that CRPA < 229 was associated with higher CD4 counts (p = 0.0082) (Fig. 4A), which was more dramatic in viremic patients (p < 0.0001) (Fig. 4B). We also looked at CD4:CD8 ratios and found a similar trend towards higher CD4:CD8 ratio in RPA < 229 group (Fig. 4C), which was significant (p = 0.0423) in the viremic group (Fig. 4D). With regards to immune activation, there was no significant difference in patients with CRPA > 229 or CRPA < 229 in either all HIV+ patients or the viremic patients group (Fig. 4E and F), although RPA > 229 had slightly higher immune activation. However, with regards to CD4 apoptosis, there was a trend towards higher apoptosis in the CRPA > 229 group in the all HIV+ patients (p = 0.0797) (Fig. 4G) but not in viremic patients (p = 0.1091) (Fig. 4H). Closer look at the viremic group revealed that one of the samples in the CRPA < 229 group was from an HHG2 patient, who had detectable viremia (8810 copies/ml) and also showed high apoptosis in CD4 cells. This patient may be harboring a dual or X4 tropic virus and when excluded from the analysis, we saw significant difference (p = 0.0351) in CD4 apoptosis between CRPA < 229 no HHG2 vs CRPA > 229 (Fig. 4I). There was no significant difference in log viremia between CRPA > 229 vs CRPA < 229 in all HIV+ (Fig. 4J) or in viremic patients (Fig. 4K).

### CRPA correlates with CD4 decline

**Figure 5:**
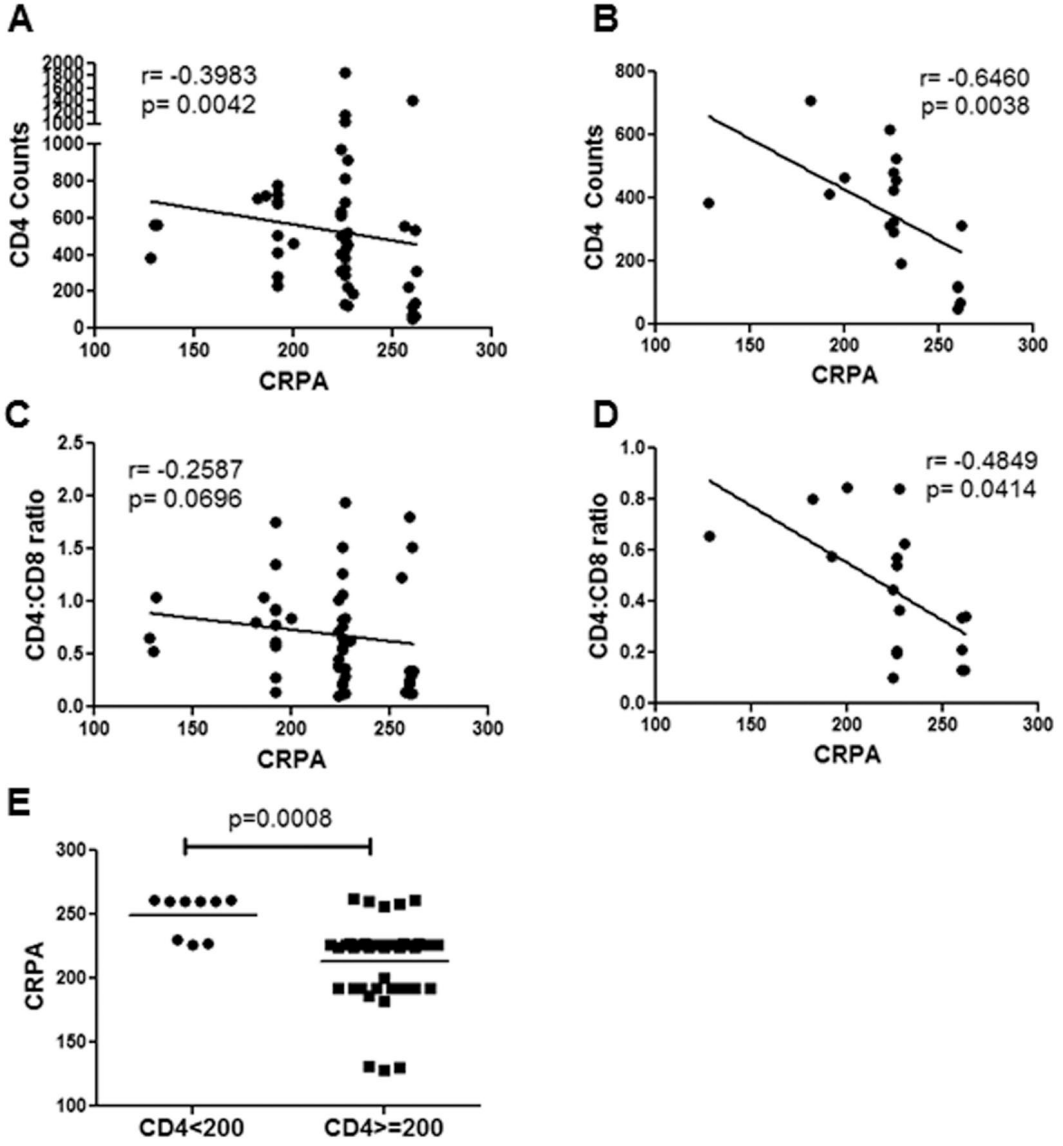
Correlation of CCR5 CRPA with CD4 decline. Correlation between CD4 counts and CCR5 CRPA in (**A**) all HIV+ or (**B**) viremic patients. Correlation of CCR5 CRPA with CD4:CD8 ratios in (**C**) all HIV+ or (**D**) viremic patients. (**E**) CRPA in patients with CD4 counts less than or more that 200/μl of blood. Spearman's correlation with linear regression was used for correlation determination. One tailed student's t-test was used for comparative statistics.

Correlation analysis and regression models are difficult with data divided into groups. Assigning CRPA scores allows us to not only conduct regression analysis but also account for both alleles in the patients. We found a strong correlation between CD4 counts and CCR5 CRPA in all HIV patients (p = 0.0042) (Fig. 5A), as well as in viremic patients (p = 0.0038) (Fig. 5B). CCR5 CRPA also correlated with CD4:CD8 ratios (Fig. 5C), being significant in viremic patients (p = 0.0414) (Fig. 5D). Finally, CRPA was significantly higher (p = 0.0008) in patients with AIDS, defined as CD4 counts less than 200/μl of blood (Fig. 5E). Linear regression models showed that the CRPA based classification correlated better with CD4 counts compared to HHC vs non HHC classification both before and after adjusting for viremia (Table 2). Taken together, these findings suggest that the CRPA based approach correlates with HIV disease progression and could be used as a parameter to predict disease outcomes.

**Table 2:** Linear regression models showing association of CCR5 promoter haplotypes (HHC and non-HHC) and Relative Promoter Activity groups (CRPA score < 229 and CRPA score >= 229) with CD4 counts, CD8 Immune Activation, CD4 apoptosis and CD4: CD8 ratio.

Outcome	Predictor: CCR5 promoter haplotypes (HHC and non-HHC)				Predictor: RPA (RPA < 229 and RPA >= 229)			
	HHC (n = 33) (mean, SD)	Non-HHC (n = 17) (mean, SD)	p-value (unadjusted)	p-value (adjusted for log-viremia)	RPA score < 229 (n = 38) (mean, SD)	RPA score >= 229 (n = 12) (mean, SD)	p-value (unadjusted)	p-value (adjusted for log-viremia)
CD4	607.39, 51.35	381.71, 341.49	**0.0348**	0.0703	598.58, 330.22	315.58, 384.08	**0.0163**	**0.0346**
CD8 Immune Activation	31.86, 19.73	38.05, 27.26	0.3621	0.9868	32.26, 20.65	39.36, 27.86	0.3456	0.9755
CD4 apoptosis	14.0, 6.41	17.6, 10.19	0.1362	0.2641	14.31, 7.25	18.05, 9.76	0.1595	0.3121
CD4: CD8 ratio	0.7, 0.47	0.66, 0.51	0.7377	0.9136	0.72, 0.44	0.59, 0.59	0.4097	0.6798

*SD = Standard deviation.

Bold = Statistically significant association.

### CRPA correlates with number of CD4+ CCR5+ T cells in the peripheral blood

**Figure 6:**
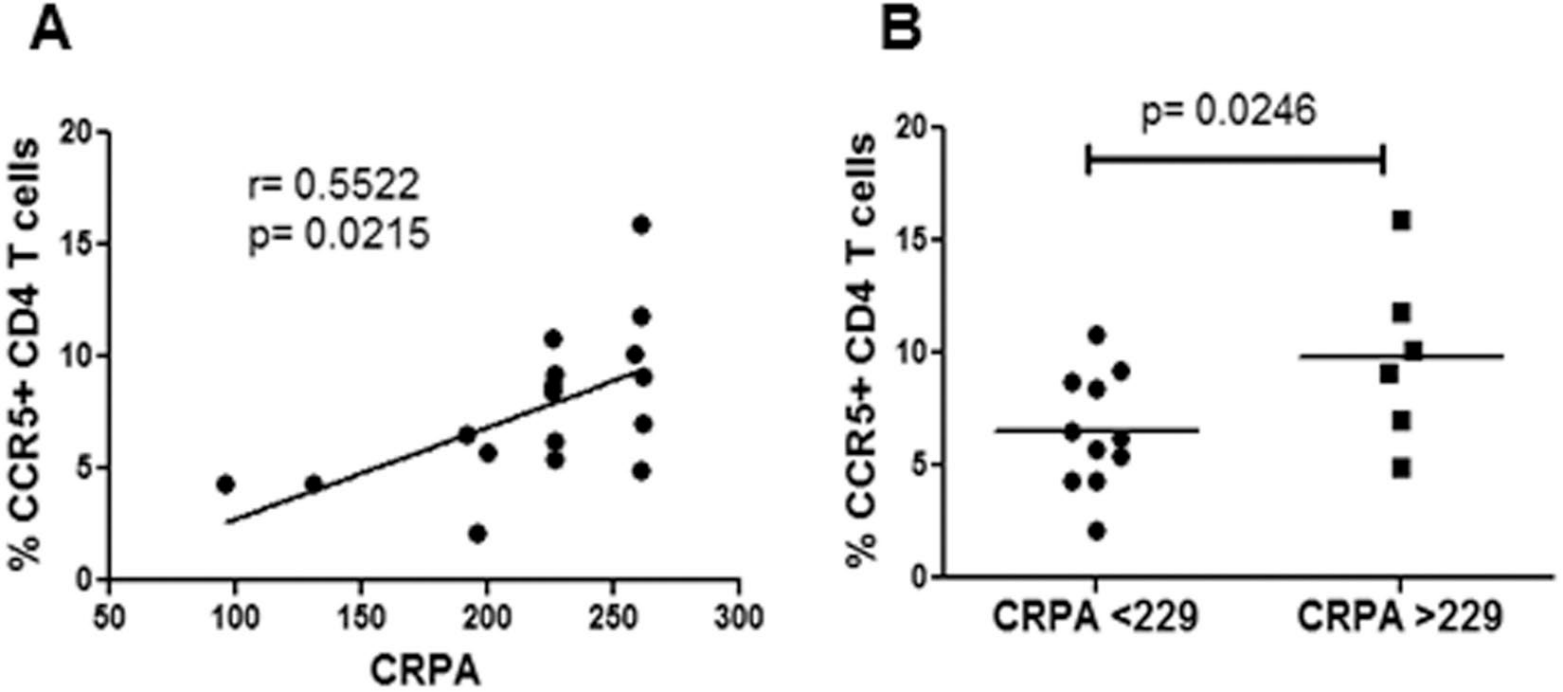
CCR5 CRPA positively correlates with number of CD4+ CCR5+ cells in the peripheral blood. (**A**) Correlation analysis of CRPA with % CCR5+ CD4+ T cells in peripheral blood of healthy controls. (**B**) Analysis of % CCR5+ CD4+ T cells in peripheral blood healthy controls after dividing the population into CRPA > 229 and CRPA < 229. Spearman's correlation with linear regression was used for correlation determination. One tailed student's t-test was used for comparative statistics.

As promoter activity is likely to affect protein expression, we next looked at the potential correlation between the CRPA methodology and cell surface expression of CCR5 in CD4+ T cells. Other groups have demonstrated that CCR5 promoter polymorphism associates with the number of CCR5+ CD4 T cells in the peripheral blood^3, 26^. Using samples from healthy controls, we found that in fact CCR5 CRPA of the individuals correlates significantly (p = 0.0215) with percent CCR5+ CD4 T cells in the peripheral blood (Fig. 6A). Also, after dividing the populations in to CRPA > 229 and CRPA < 229 groupings, we found that there was a significant difference (p = 0.0246) in CCR5+ cells between the groups (Fig. 6B). These data are in line with results of others who have shown a correlation between CCR5 polymorphisms and CCR5 expression^3, 12, 28^. However, we show here that assigning a CRPA value to each sample can be an alternate method for correlating polymorphisms/genotype with different phenotypes.

### R5 virus infected viremic patients show correlation between CCR5 CRPA and CD4 apoptosis and CD4 decline

**Figure 7:**
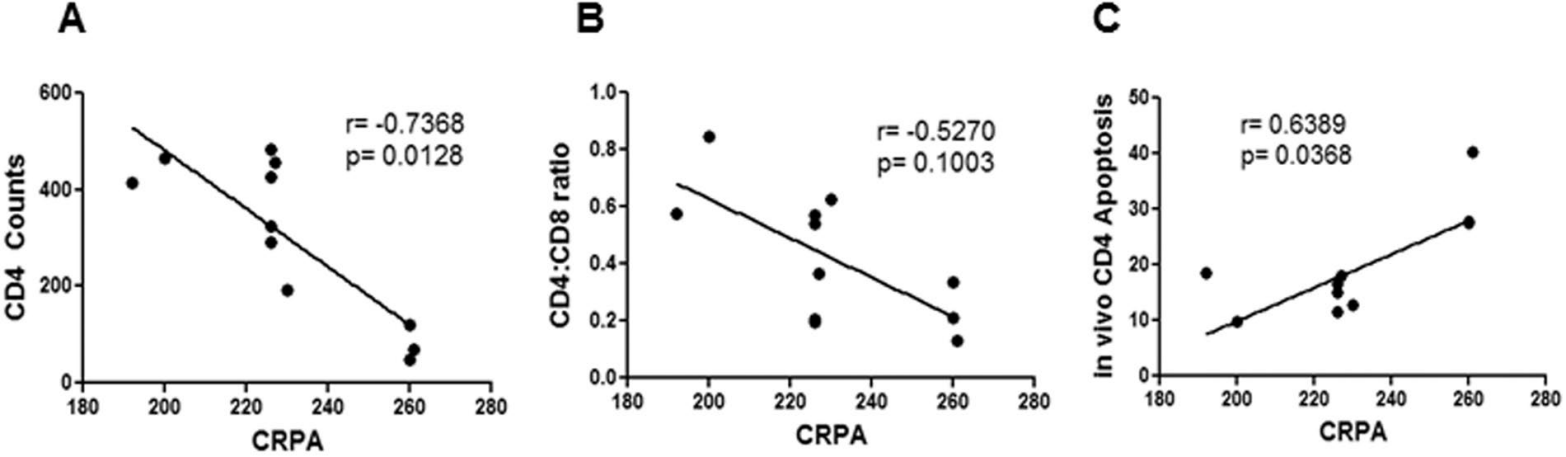
Correlation between CCR5 CRPA, CD4 apoptosis and CD4 decline in CCR5 tropic HIV infected viremic patients. Full length functional Env glycoproteins cloned from 11 viremic patients in the HIV patient cohort were previously found to be CCR5 tropic. Correlation analysis of CCR5 CRPA with (**A**) CD4 counts (**B**) CD4:CD8 ratio (**C**) *in vivo* CD4 apoptosis in these R5 virus harboring patients. Spearman's correlation with linear regression was used for all correlation determination.

We next asked whether we could get additional information regarding the mechanism behind the correlation between CCR5 polymorphisms and HIV pathogenesis. CCR5 is the major co-receptor for HIV and is directly involved in binding to the viral Env glycoprotein. Env-CCR5 interactions are fundamental to both viral entry and pathogenesis. In fact, the role of CCR5 binding by Env in HIV mediated bystander apoptosis has been demonstrated^27, 29^. We previously cloned full length Env glycoproteins from 11 of the high viremic patients (>10,000 copies/ml) in our cohort and found that all of these Envs were CCR5 tropic^24^. We hence asked whether in individuals with high viremia, harboring R5 tropic virus, if there was a correlation between CCR5 CRPA and CD4 decline and/or CD4 apoptosis. As shown in Fig. 7A there was a significant correlation (p = 0.0128) between CD4 counts and CCR5 CRPA and a strong trend between CCR5 CRPA and CD4:CD8 ratios (Fig. 7B). Moreover, a significant correlation (p = 0.0368) was found between CD4 apoptosis and CCR5 CRPA (Fig. 7C) consistent with data above. These findings suggest that the correlation between CCR5 CRPA and CD4 counts could in part be mediated by the susceptibility of cells to Env mediated bystander apoptosis.

## Discussion

The correlation between CCR5 promoter polymorphisms in HIV disease progression has been extensively studied and a strong relationship between certain CCR5 promoter haplotypes and disease progression has been demonstrated^1, 6, 15, 21, 30^. While these correlations have often been associated with CCR5 levels, the mechanism behind the role of CCR5 promoter polymorphisms and HIV disease progression remains less certain.

Gonzalez *et al*. demonstrated that HHE haplotype is associated with accelerated progression to AIDS in Caucasian population, while HHC may be associated with delayed progression^6^. Although HHC and HHE are two of the most common haplotypes, as was also confirmed in our study, there are other haplotypes that need to be investigated. With the frequency of other haplotypes being low in many cohorts it becomes difficult to undertake these studies based on genotypic classification alone. Also, it is imperative that allele pairs be accounted for in these studies. Since CCR5 promoter polymorphisms may influence CCR5 protein levels, and thus affect disease progression^28, 31, 32^, we investigated whether promoter activity could be used as a surrogate for CCR5 levels and consequently disease progression in HIV patients.

We established the RPA of each haplotype using an *in vitro* gene reporter assay. After normalizing to HHA, the ancestral haplotype^5^, we assigned numerical values to each promoter haplotype. Similarly, each patient could be assigned a CRPA based on the sum of the RPAs of the 2 alleles of each individual. This method converts genotypic information into a numerical score based on aggregate promoter activity of the allele pair. Using this approach we could now stratify our population based on functional CRPA scores, and not just on allele combinations. With this method we found that HHE, HHF and HHG had similar promoter activity that was about 130% of the ancestral HHA haplotype, and that HHC activity was slightly lower than HHA at 96%. We were able to stratify our population into two groups of patients, those with CRPA < 229 or > 229. Notably, patients with HHE/HHE, HHE/HHF or HHE/HHG1 belonged to the high CRPA group and those with HHC and other low activity haplotypes like HHA, HHB or HHD were in the low group. Using this strategy, we found a correlation between CD4 counts and CRPA values, with the cutoff between CRPA < 229 vs > 229.

**Figure 8:**
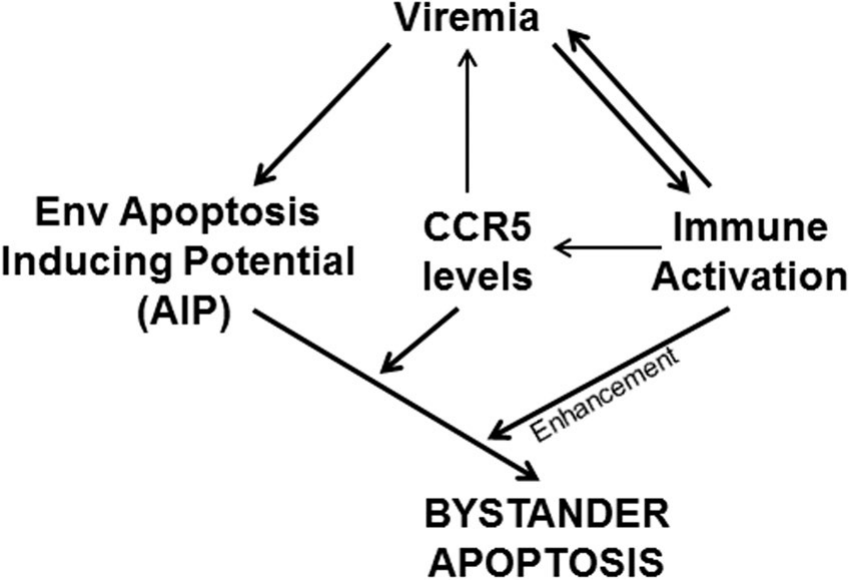
Model of HIV pathogenesis proposing a role of Env glycoprotein phenotype (AIP), viremia, immune activation and CCR5 levels in collectively determining CD4 bystander apoptosis. The model incorporates CCR5 levels at the center as a major factor which may regulate both plasma viral load and apoptosis mediated by Env glycoprotein leading to CD4 decline.

Interestingly, we found that the differences between low and high CRPA groups were more significant when considering viremic patients (≥100 virus copies/ml of viral RNA) compared to the total population. As CCR5 is the major co-receptor for HIV and the viral Env glycoprotein directly binds to it, it is not surprising that the effects are more relevant in the presence of active virus replication/viremic patients. At the same time, we also found that differences between CRPA groups showed a strong trend in terms of CD4 apoptosis. We and others have shown that CD4 apoptosis correlates with CD4 decline in HIV patients^24, 33–35^. Also, binding of HIV Env to CCR5 is required for bystander apoptosis induction^29, 36^. We have also shown that levels of CCR5 on cell surface determines bystander apoptosis of cells via HIV Env, with higher CCR5 expression associated with increased bystander apoptosis *in vitro* ^27, 37^. The higher apoptosis in the CRPA > 229 group is consistent with this idea. Furthermore, within the group of high viremic patients where we were successful in cloning full length CCR5 tropic Envs, we found a strong correlation between CCR5 CRPA and CD4 apoptosis. Taken together, these data suggest that higher CCR5 promoter activity and consequently higher CCR5 expression may be related to higher CD4 apoptosis mediated by primary R5 Envs (Fig. 8).

While bystander apoptosis is one the factors in HIV pathogenesis, it is clear from recent studies^24, 38^ that HIV pathogenesis is multifactorial. In a model presented in our previous study^24^ we proposed a role for the Env glycoprotein phenotype, viremia and immune activation in collectively mediating CD4 bystander apoptosis. Based on the findings from this study, inclusion of CCR5 promoter polymorphisms in this model is justified (Fig. 8). This is because levels of CCR5 can affect other HIV pathogenesis regulating factors as well as be altered by them. We know that increased cell surface CCR5 levels would likely support higher virus replication^37^, as is clear from the lower viremia in CCR5Δ32 heterozygous individuals^39^. The strong association between viremia and immune activation in HIV infections suggests that higher virus replication in CCR5 WT individuals may be related to higher immune activation. A trend towards higher immune activation in our samples is indicative of this phenomenon. Recently, Gornalusse *et al*. have shown that immune activation can directly affect CCR5 promoter activity and alter cell surface CCR5 expression^32^. Finally, levels of CCR5 can affect HIV-mediated CD4 loss as seen *in vitro* by us^27^ as well as in SCID-hu mice studies by Scoggins *et al*.^40^. Interestingly, a role of anti CCR5 antibodies in mediating CCR5 downregulation has been demonstrated in some long term non progressors (LTNP)^41^, further supporting a role for CCR5 levels in disease progression. Hence, a new and more extensive model of HIV pathogenesis can now be presented that incorporates CCR5 levels regulated via CCR5 gene and promoter polymorphisms (Fig. 8).

Overall, we have quantified the activity of different CCR5 promoter haplotypes, and used this information to assign each individual a CRPA score based on their allele pair. Our results yielded strong correlation between CRPA and CD4 levels in HIV+ patients. The correlation of CD4 T cell apoptosis with CCR5 polymorphism is also shown for the first time in our study. Hence our study provides a mechanistic explanation to the relationship between CCR5 polymorphisms and CD4 loss/disease progression. Use of the CRPA strategy for determining disease outcome in HIV patients needs to be further validated in longitudinal studies as well as in larger sample sizes. We anticipate that this methodology can be used for prognosis and for improved design of anti-HIV therapeutic regimens.

## Methods

### Ethical Statement

The study was reviewed and approved by the Texas Tech University Health Sciences Center, El Paso, Institutional Review Board (IRB) and all experiments were performed in accordance with relevant guidelines and regulations. The study design was cross-sectional and the study number recorded as IRB# E12092, approval date 07/31/2012. All participants were provided with written and oral information about the study. Written informed consent of all study participants in accordance with the institutional policy was documented. In accordance with institutional IRB guidelines, all participants were identified by coded numbers to assure anonymity and all patient records kept confidential.

### Patient population

Fifty HIV-infected individuals and 28 healthy controls were recruited from the outpatient HIV clinic at the Texas Tech University Health Sciences Center at El Paso. The mean age of the HIV+ patient population was 37.9 ± 11.9. The HIV group comprised of 9 females (18.0%) and 41 males (82.0%). The study was cross sectional consisting of patients at different stages of the disease. Viremic patients were defined as those having ≥100 copies of viral RNA. An age matched healthy population control group (n = 28) was also recruited from the same geographical location and comprised of 9 females (32%) and 19 males (68%). The mean age of the healthy control group was 34.5 ± 10.12. Further details of patient population including CD4 counts, viral load and HAART status have been described previously^24^.

### Sample collection and storage

Each patient provided a 20 ml blood sample that was separated into plasma and cellular components using Ficoll based separation. Genomic DNA was extracted from whole blood samples using the QIAamp DNA Blood Mini kit (Qiagen). All components from the sample including plasma, cells and DNA were aliquoted and stored at −70 °C till further analysis.

### CCR5 gene and promoter polymorphisms

Genomic DNA isolated from whole blood was used for PCR amplification of CCR5 promoter region or for CCR5Δ32 genotype determination. CCR5Δ32 genotype was determined by PCR amplification using primers spanning the 32 bp deletion as described by Kaur *et al*.^42^. Individuals with the Δ32 allele showed a 32 bp smaller PCR fragment compared to wild type. CCR5 promoter polymorphisms were determined by PCR amplification of CCR5 promoter region from genomic DNA using Phusion high fidelity PCR kit (New England Biosciences) followed by Sanger’s sequencing. Sequences were analyzed using the DNA STAR software and polymorphisms identified based on sequence peaks.

### Generation of CCR5 promoter haplotypes

CCR5 promoter regions from HHC or HHG homozygous individuals were PCR amplified using specific primers spanning the region −3139 to −1317 (CCR5 long promoter) or −2761 to −1814 (CCR5 short promoter) based on numbering by Mummidi *et al*.^5^ and cloned into TA cloning kit (Invitrogen). Subsequently, the promoter fragment was digested with Kpn-1 and Xho-1 and sub cloned into pGL-3 basic vector (Promega) upstream of the luciferase gene. Site directed mutagenesis with Quick Change Site Directed Mutagenesis Kit (Stratagene) was used to generate the haplotypes HHA, HHB, HHD, HHE and HHF.

### CCR5 promoter activity determination

CCR5 promoter activity was determined in 293 T cells by transfecting with plasmid DNA (0.3 μg/well of a 96-well plate) from each CCR5 gene promoter haplotype (HHA, HHB, HHC, HHD, HHE, HHF and HHG) using the TurboFect™ Transfection Reagent (Fisher Scientific). Promoter activity was determined 48 h later by measuring luciferase activity in cell lysates using Brite Lite Substrate (Perkin Elmer). Relative promoter activity (RPA) of each haplotype was calculated after normalizing to HHA, the ancestral allele. Each HIV patient was given a CRPA score based on their two alleles.

### Immunostaining

Staining of cells for different immune markers has been described previously^24^. Briefly, lymphocytes isolated from the blood samples obtained from HIV-infected or normal patients were stained for cell surface markers using specific antibodies (Supplementary Figure 2). The immune activation panel consisted of antibodies CD3-Cy7, CD4-Tx red, CD8-APC (Beckman Coulter) along with immune activation markers CD38 PE and HLA-DR FITC (BD Biosciences). The apoptosis panel comprised of the following antibodies CD3-Cy7, CD4- Tx Red, CD8-APC (Beckman Coulter), CCR5 PE (BD Biosciences) along with apoptosis marker CaspACE FITC-VAD-FMK (Promega). Stained cells were washed and fixed using Cytofix reagent (Beckman coulter) and acquired on a 10 color Beckman Coulter Gallios flow cytometer. At least 20,000 events for each sample were acquired. Data was analyzed using FlowJo software (Tree Star). Cells were first gated on CD3+ population and immune activation/apoptosis on CD4+ and CD8+ T cell subsets determined.

### Statistical analysis

Data were analyzed using GraphPad Prism (GraphPad Software, Inc, La Jolla, CA) and SAS 9.3 (SAS Institute, Inc., Cary, North Carolina). Differences between groups were assessed using the two-sample t-test; either one sided or two sided based on the hypothesis. All p values were considered significant at p < 0.05. Spearman’s correlation with linear regression was used for all correlation determination using the GraphPad Prism Software. The association of CCR5 haplotypes (HHC and non-HHC) and CRPA groups (CRPA < 229 and CRPA > = 229) with CD4 counts, CD8 immune activation, CD4 apoptosis and CD4:CD8 ratio was determined using linear regression models, unadjusted and adjusted for viremia.

## Electronic supplementary material

Supplementry information
