# Supplementary material for: CCR5 promoter activity correlates with HIV disease progression by regulating CCR5 cell surface expression and CD4 T cell apoptosis
Source: Sci Rep. 2017 Mar 22;7:232. doi: 10.1038/s41598-017-00192-x (PMC5427887; doi:10.1038/s41598-017-00192-x)
Supplement: Supplementary file 1 — Supplementry information [file 41598_2017_192_MOESM1_ESM.pdf]

CCR5 promoter activity correlates with HIV disease progression by regulating CCR5 cell surface expression and CD4 T cell apoptosis.

Anjali Joshi, Erin B. Punke, Melina Sedano, Bethany Beauchamp, Rima Patel, Cassady Hossenlopp, Ogechika K. Alozie, Jayanta Gupta, Debabrata Mukherjee and Himanshu Garg.

Supplementary Table 1: SNPs identified in the human CCR5 promoter region.

Haplotype	58755	58934	59029	59353	59356	59402	59653	CCR5 Delta 32
**HHA**	A	G	G	T	C	A	C	WT
**HHB**	A	T	G	T	C	A	C	WT
**HHC**	A	T	G	T	C	G	C	WT
**HHD**	A	T	G	T	T	A	C	WT
**HHE**	A	G	A	C	C	A	C	WT
**HHF**	A	G	A	C	C	A	T	WT
**HHG1**	G	G	A	C	C	A	C	WT
**HHG2**	G	G	A	C	C	A	C	Δ32

CCR5 haplotypes HHA-HHG based on seven SNPs in the promoter region.
The HHG2 haplotype is in linkage disequilibrium with CCR5Δ32 genotype.

Supplementary Table 2: Frequency of different CCR5 promoter SNPs in the HIV+ population (HP) and normal controls (NR).

		Population (frequency)	
**SNP**	**Genotype**	**HP**	**NR**
**G58755A**	GG	2 (0.040)	0 (0.000)
	GA	9 (0.180)	6 (0.214)
	AA	39 (0.780)	22 (0.786)
**T58934G**	TT	10 (0.200)	3 (0.107)
	TG	24 (0.480)	13 (0.464)
	GG	16 (0.320)	12 (0.429)
**G59029A**	GG	12 (0.240)	5 (0.179)
	GA	24 (0.480)	12 (0.429)
	AA	14 (0.280)	11 (0.393)
**T59353C**	TT	12 (0.240)	5 (0.179)
	TC	24 (0.480)	12 (0.429)
	CC	14 (0.280)	11 (0.393)
**C59356T**	CC	48 (0.960)	28 (1.000)
	CT	2 (0.040)	0 (0.000)
	TT	0 (0.00)	0 (0.000)
**G59402A**	GG	9 (0.180)	3 (0.107)
	GA	24 (0.480)	13 (0.464)
	AA	17 (0.340)	12 (0.429)
**C59653T**	CC	40 (0.800)	16 (0.571)
	CT	9 (0.180)	10 (0.357)
	TT	1 (0.020)	2 (0.071)

Supplementary Table 3: Combined Relative Promoter Activity (CRPA) score in the HIV+ population.

	N	%	CRPA
**HHC/HHE**	12	24	226
**HHC/HHC**	9	18	192
**HHC/HHG1**	6	12	224
**HHC/HHF**	5	10	227
**HHE/HHE**	5	10	260
**HHE/HHF**	3	5	261
**HHG2/HHG1**	1	2	128
**HHG2/HHE**	1	2	130
**HHG2/HHF**	1	2	131
**HHC/HHD**	1	2	182
**HHA/HHD**	1	2	186
**HHA/HHA**	1	2	200
**HHA/HHE**	1	2	230
**HHG1/HHG1**	1	2	256
**HHE/HHG1**	1	2	258
**HHF/HHF**	1	2	262

The CRPA is based on the sum of the RPA of each of the CCR5 allele pair. Frequency (N) and percent (%) of each allele pair is also shown.

Supplementary Table 4: Characteristics of viremic and non-viremic HIV patients in the study.

Variable	*Non-Viremic (<100 copies of viral RNA) (n=32)*	*Viremic (>100 copies of viral RNA) (n=18)*	*P value*
**CD4 counts**	633.5 ± 69.96	347.9 ± 44.00	0.006
**CD4:CD8 Ratio**	0.8267 ± 0.92	0.4403 ± 0.05	0.0049
**CD4 activation**	9.05 ± 1.31	23.92 ± 4.55	0.0003
**CD8 activation**	21.23 ± 2.32	56.61 ± 4.04	<0.0001
**CD4 Apoptosis**	12.74 ± 1.00	19.59 ± 2.27	0.0027
**Log Viremia**	1.357 ± 0.02	4.186 ± 0.26	<0.0001

Full length Envs (Gene Bank Accession No.) were cloned for HP003 (KP754463), HP013 (KP754464), HP015 (KP754465), HP022 (KP754466), HP024 (KP754467), HP025 (KP754468), HP029 (KP754469), HP038 (KP754470), HP042 (KP754471), HP043 (KP754472), HP051 (KP754473).

A

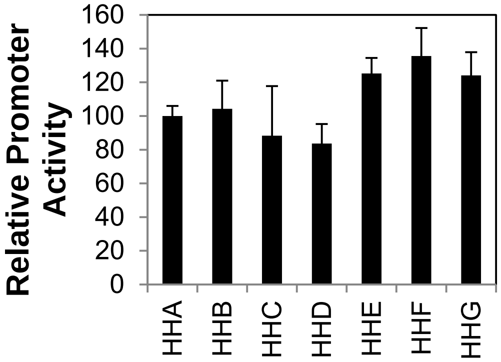

B

Haplotype	RPA
**HHA**	100
**HHB**	104
**HHC**	88
**HHD**	84
**HHE**	125
**HHF**	136
**HHG1**	124
**HHG2**	0

Supplementary Figure 1: Transcriptional activity of different CCR5-short promoter haplotypes: (A) 293T cells were transfected with luciferase gene reporter plasmid DNA containing CCR5 promoter regions (-2761 to -1814) from haplotypes HHA-HHG. Promoter activity was determined 48h later by measuring luciferase activity in cell lysates. Data was normalized to HHA, the ancestral haplotype. **(B)** Relative promoter activity (RPA) of each CCR5 promoter haplotype after normalizing to the ancestral allele HHA.

A

Marker	Manufacturer	Reagent	Cat No.	Amount/Sample
**CD3**	BD Pharmingen	PE-Cy7 Mouse Anti-Human CD3 clone SK-7	557851	5µl
**CD4**	BD Horizon	PE-CF594 Mouse Anti-Human CD4 clone RPA-T4	562281	5µl
**CD8**	BD Pharmingen	APC Mouse Anti-Human CD8	555369	5µl
**CD38**	BD Pharmingen	PE Mouse Anti-Human CD38	555460	10µl
**HLA-DR**	BD Pharmingen	FITC Mouse Anti-Human HLA-DR	555811	10µl
**CCR5/CD195**	BD Pharmingen	PE Mouse Anti-Human CD195	55593	10µl
**Caspase**	Promega	CaspACE FITC-VAD-FMK In Situ Marker	G7462	10µM

B

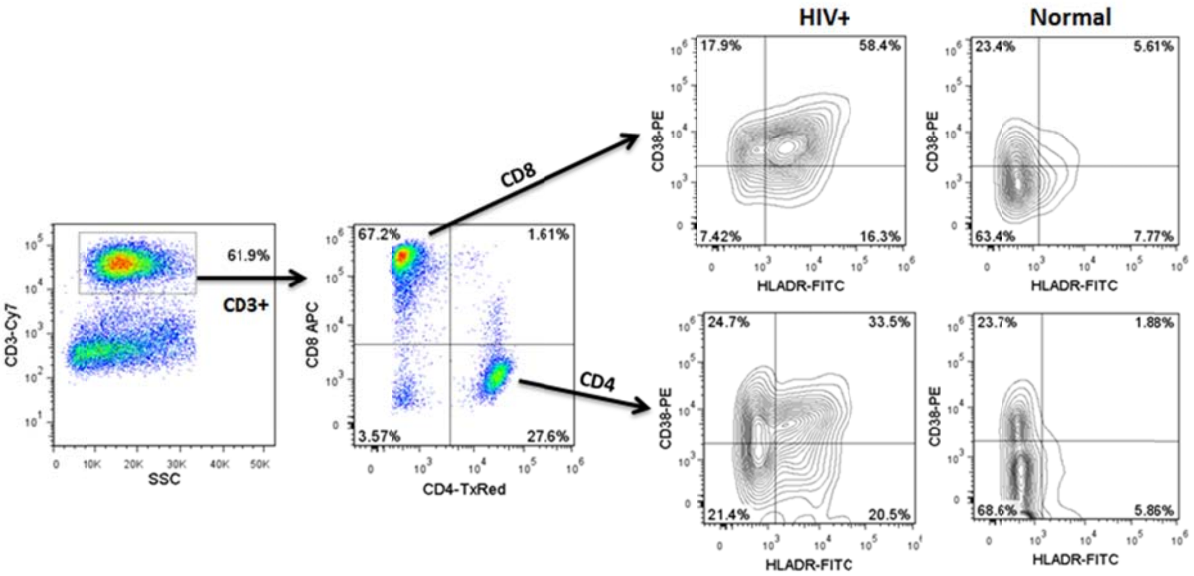

Supplementary Figure 2: Immunostaining of patient samples and gating strategy for flow cytometry analysis for determining immune activation. (A) List of antibodies and concentrations used in the study for immunostaining of PBMCs derived from HIV infected patients and normal controls. **(B)** Gating strategy for determination of immune activation in CD4 and CD8 cells in HIV+ patients and normal controls. Cells were first gated on CD3+ population and immune activation on CD4+ and CD8+ T cell subsets determined via HLA-DR staining. Representative staining panel and gating strategy form an HIV infected individual and normal control is shown.
